# Medical Student’s Perception of Dementia Assessment and Management Among Rural Veterans

**DOI:** 10.1093/geroni/igaa057.2887

**Published:** 2020-12-16

**Authors:** Prasad Padala, Jessica Stovall, Matthew Kern, Jeremy Curtis, Eugenia Boozer, Shelly Lensing, Kalpana Padala

**Affiliations:** 1 Central Arkansas Veterans Healthcare System, Little Rock, Arkansas, United States; 2 Central Arkansas Veterans Healthcare System, North Little Rock, Arkansas, United States; 3 University of Arkansas for Medical Sciences, Little Rock, Arkansas, United States

## Abstract

Background: Rural Veterans rely on their caregivers, case managers and primary care providers for dementia management. Providers of such patients need to work closely with caregivers, know the local dementia resources and be comfortable with the multiple facets of dementia assessment and management. Unfortunately, medical students are not particularly well trained in these aspects and huge knowledge gaps exist. The goal was to study the impact of a multi-component, experiential, brief curriculum on attitudes of dementia care. Methods: 108 medical students participated in a curriculum including didactics, clinical, and team-based learning followed by pre-post assessments. Results: Student’s perception of their ability to assess multiple facets of dementia such as behaviors, caregiver burden, and cognition improved significantly (p<0.001). Students’ perception of the role of social worker improved significantly (p=0.002). Conclusion: An interdisciplinary curriculum, improved medical students’ perception of their ability to assess for dementia in a cohort of predominantly rural Veterans.

